# From Hormones to Harvests: A Pathway to Strengthening Plant Resilience for Achieving Sustainable Development Goals

**DOI:** 10.3390/plants14152322

**Published:** 2025-07-27

**Authors:** Dipayan Das, Hamdy Kashtoh, Jibanjyoti Panda, Sarvesh Rustagi, Yugal Kishore Mohanta, Niraj Singh, Kwang-Hyun Baek

**Affiliations:** 1 Department of Microbiology, The Assam Royal Global University, Guwahati 781035, Assam, India; ddmicrobiology@gmail.com; 2 Department of Biotechnology, Yeungnam University, Gyeongsan 38541, Gyeongsangbuk-do, Republic of Korea; hamdy_kashtoh@ynu.ac.kr; 3 Bioresource and Traditional Knowledge Laboratory, Department of Wildlife and Biodiversity Conservation, Maharaja Sriram Chandra Bhanja Deo University, Baripada 757003, Odisha, India; jibanjyotipanda83@gmail.com; 4 Department of Food Technology, School of Agriculture, Maya Devi University, Dehradun 248011, Uttarakhand, India; sarveshrustagi@gmail.com; 5 Nano-Biotechnology and Translational Knowledge Laboratory, Department of Applied Biology, University of Science and Technology Meghalaya, Baridua 793101, Meghalaya, India; ykmohanta@gmail.com; 6 Centre for Herbal Pharmacology and Environmental Sustainability, Chettinad Hospital and Research Institute, Chettinad Academy of Research and Education, Kelambakkam 603103, Tamil Nadu, India

**Keywords:** phytohormone, crosstalk, abiotic response, SDG, food security, climate change, adaptation

## Abstract

The worldwide agriculture industry is facing increasing problems due to rapid population increase and increasingly unfavorable weather patterns. In order to reach the projected food production targets, which are essential for guaranteeing global food security, innovative and sustainable agricultural methods must be adopted. Conventional approaches, including traditional breeding procedures, often cannot handle the complex and simultaneous effects of biotic pressures such as pest infestations, disease attacks, and nutritional imbalances, as well as abiotic stresses including heat, salt, drought, and heavy metal toxicity. Applying phytohormonal approaches, particularly those involving hormonal crosstalk, presents a viable way to increase crop resilience in this context. Abscisic acid (ABA), gibberellins (GAs), auxin, cytokinins, salicylic acid (SA), jasmonic acid (JA), ethylene, and GA are among the plant hormones that control plant stress responses. In order to precisely respond to a range of environmental stimuli, these hormones allow plants to control gene expression, signal transduction, and physiological adaptation through intricate networks of antagonistic and constructive interactions. This review focuses on how the principal hormonal signaling pathways (in particular, ABA-ET, ABA-JA, JA-SA, and ABA-auxin) intricately interact and how they affect the plant stress response. For example, ABA-driven drought tolerance controls immunological responses and stomatal behavior through antagonistic interactions with ET and SA, while using SnRK2 kinases to activate genes that react to stress. Similarly, the transcription factor MYC2 is an essential node in ABA–JA crosstalk and mediates the integration of defense and drought signals. Plants’ complex hormonal crosstalk networks are an example of a precisely calibrated regulatory system that strikes a balance between growth and abiotic stress adaptation. ABA, JA, SA, ethylene, auxin, cytokinin, GA, and BR are examples of central nodes that interact dynamically and context-specifically to modify signal transduction, rewire gene expression, and change physiological outcomes. To engineer stress-resilient crops in the face of shifting environmental challenges, a systems-level view of these pathways is provided by a combination of enrichment analyses and STRING-based interaction mapping. These hormonal interactions are directly related to the United Nations Sustainable Development Goals (SDGs), particularly SDGs 2 (Zero Hunger), 12 (Responsible Consumption and Production), and 13 (Climate Action). This review emphasizes the potential of biotechnologies to use hormone signaling to improve agricultural performance and sustainability by uncovering the molecular foundations of hormonal crosstalk. Increasing our understanding of these pathways presents a strategic opportunity to increase crop resilience, reduce environmental degradation, and secure food systems in the face of increasing climate unpredictability.

## 1. Introduction

The worldwide agricultural environment is changing dramatically as the dual stresses of population growth and climate change increase. The United Nations predicts that the world’s population will exceed 9 billion by 2050. This population transition demands a stunning 70% rise in global agricultural output to ensure universal food security, according to Francini and Sebastiani [1]. However, this goal is becoming more difficult in light of the increasingly unfavorable weather conditions that impair crop output and threaten to undermine global food security. Abiotic challenges such as salt, drought, heatwaves, and heavy metal toxicity, along with biotic concerns such as pest invasions, plant diseases, and nutritional deficits, present a serious challenge to traditional agricultural approaches [2,3,4,5]. These rising challenges underscore the urgent need for innovative solutions aligned with the United Nations Sustainable Development Goals (SDGs), particularly SDG 2 (Zero Hunger), SDG 13 (Climate Action), and SDG 12 (Responsible Consumption and Production). The relationship between SDG 2: Zero Hunger and SDG 12: Responsible Consumption and Production creates a complex web of issues arising from their shared commitment to environmental sustainability and resource management [6]. The convergence of sustainable agriculture and responsible production is reflected in both goals, with sustainable agricultural techniques aiming to maximize food production while minimizing environmental impact [7].

This paradox promotes SDG 2 by increasing food security while protecting valuable natural resources. Similarly, SDG 12 emphasizes environmentally friendly production, decreased emissions, and waste prevention, aligning with both goals in promoting a greener footprint [8]. Consequently, SDG 2 and SDG 13 both call for adaptation measures to ensure food security in the face of climate change. Dörg ő et al. emphasize the need to maintain access to food in the face of climate change [9]. Conventional breeding approaches, while historically effective in boosting yield and resistance qualities, are no longer adequate to fulfill the complex demands of the twenty-first century. The complexities of stress tolerance, which are mediated by extensive gene–environment interactions and regulatory processes, have restricted the effectiveness and predictability of traditional methods. In this context, using plant hormones (phytohormones) as instruments to increase resilience and productivity appears to be a promising and long-term strategy. Phytohormones are chemical messengers produced by plants that regulate their growth, development, and stress tolerance. These compounds, which include abscisic acid (ABA), gibberellins (GAs), ethylene, salicylic acid (SA), jasmonic acid (JA), and cytokinins, coordinate a wide range of physiological responses that allow plants to survive and reproduce under adverse conditions [10,11]. For example, ABA plays a crucial role in mediating drought stress responses by regulating stomatal closure and water-use efficiency, whereas GA and ethylene influence responses to severe temperatures and heavy metal exposure. These hormones cooperate to regulate stress reactions and developmental processes through a dynamic interplay of antagonistic and synergistic interactions known as hormonal crosstalk [12,13].

Recent studies have shown that phytohormones can improve plant defenses and produce consistent yields even under challenging circumstances [14]. Research on model and crop species, including important horticultural and staple crops, demonstrates that stress tolerance and productivity can be significantly increased by genetically or exogenously modifying hormonal pathways [15,16]. These results provide a solid scientific basis for applying methods based on phytohormones to sustainable agriculture. For instance, it has been shown that exogenous ABA or its analogs enhance drought resilience, and treatments with JA have increased pest resistance in cereals and legumes [17]. By reducing crop losses and dependence on artificial agrochemicals, such strategies promote ecologically friendly farming methods. Hormonal crosstalk can revolutionize plant stress management and directly support many SDGs if it is understood and used. Enhancing yield stability and crop resilience can help achieve SDG 2, which aims to end hunger and advance food security for all. Reducing crop failure brought on by environmental stresses also advances SDG 13, which demands swift action to address climate change and its effects [8]. Phytohormone-driven technologies enable crops to endure extreme climate events, adapt to degraded soils, and thrive in water-scarce conditions in an era of rising temperatures, unpredictable rainfall, and land degradation.

Furthermore, using hormone-mediated resilience mechanisms in agricultural techniques advances SDG 12, which promotes sustainable patterns of production and consumption. Reducing reliance on chemical fertilizers and pesticides is consistent with the tenets of the circular economy, which also safeguard biodiversity and ecosystem services. Phytohormone-based interventions fortify plants’ defense mechanisms, offering a low-input, high-impact alternative to sustainable intensification that achieves production goals while preserving ecological balance.

Despite these encouraging possibilities, an integrated strategy involving fundamental research, translational science, and policy frameworks is required for successful large-scale implementation. To develop hormone-based products, promote their safe and efficient use, and ensure equitable access to these innovations, stakeholders, including scientists, farmers, legislators, and agro-industrial partners, must collaborate. In order to disseminate information and promote adoption, farmer training and extension services are essential, particularly for smallholder communities, who are disproportionately vulnerable to climate shocks. The move from hormones to harvests represents a paradigm change in contemporary agriculture, emphasizing plant resilience as the cornerstone of world food security. A sustainable agricultural future that feeds the world without destroying the environment can be achieved by tying plant hormone research and use to the Sustainable Development Goals. Incorporating hormonal crosstalk into crop improvement techniques will be essential to ensuring that agriculture continues to flourish, feeding populations, preserving ecosystems, and achieving sustainable development as climate variability rises and resource constraints worsen.

## 2. Research Gap

Despite tremendous progress in understanding the phytohormonal regulation of plant responses to abiotic stresses, numerous key research gaps remain, preventing this information from being fully translated into practical, field-level applications. Extensive study over the last decade has revealed the importance of phytohormones (auxin, ABA, SA, JA, ethylene, and GA) in regulating plant responses to drought, salinity, heat, and cold stress. The complexity of hormonal crosstalk and dynamic modulation under multifactorial stress situations is still scarcely characterized in real-world agroecosystems [18]. One prominent gap is the context-dependency of hormonal responses, which can cause the same hormone to have distinct effects depending on the species, developmental stage, and environmental situation. The majority of current research focuses on model plants or selected high-yielding cultivars, with less emphasis on orphan or underutilized crops, which are crucial for food security in marginal areas [19]. This results in an insufficient understanding and restricts the scalability of phytohormone-based therapeutics. Whereas transcriptome and metabolomic studies have shown hormone-induced regulatory networks, the spatiotemporal dynamics of these pathways under simultaneous abiotic stress conditions have received less attention [20]. Plants in the field are frequently exposed to drought, salinity, and heat, but most research is conducted under single-stress conditions that fail to simulate genuine environmental complexity [21]. As a result, a systems-level strategy that combines many layers of omics with phenotypic plasticity in poly-stress situations is urgently required. Another unexplored topic is the creation of delivery mechanisms for exogenous hormone administration. While foliar sprays and seed priming have shown potential, the optimization of hormone formulation, dose, timing, and carrier systems for diverse crops and climates is variable and unstandardized [22]. There is a scarcity of field validation and long-term agronomic trials to determine the long-term viability and environmental safety of repeated hormone treatments. This disparity reduces regulatory approval and farmer implementation, particularly in resource-constrained areas. While the emphasis of this review is primarily on crop plants, it is important to recognize that these challenges and research needs are equally pertinent to woody perennials, especially fruit trees and vines. Their perennial growth habits introduce additional layers of complexity in terms of hormonal regulation, stress memory, and seasonal responses, which require dedicated investigation. Bridging these research gaps is critical to maximizing the potential of phytohormonal methods for resilient agriculture in accordance with the SDGs.

## 3. Phytohormones in Abiotic Stress Responses in Plants

**Figure 1:**
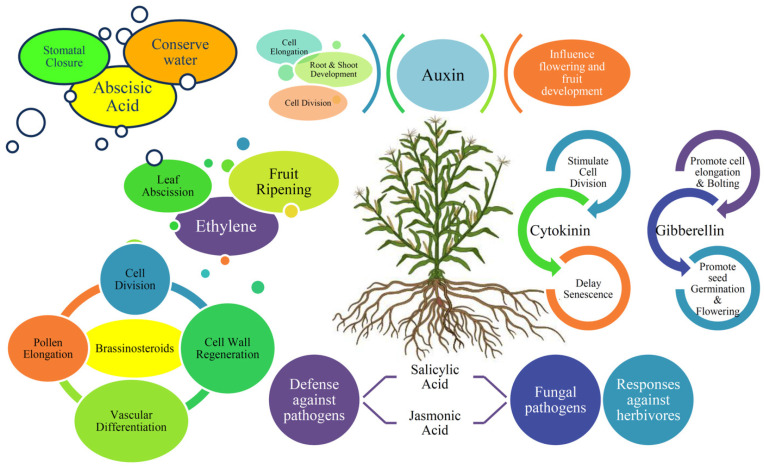
Fundamental hormonal response under biotic and abiotic stress.

Abiotic stresses such as drought, salinity, extreme temperatures, and nutrient imbalances severely limit plant growth and productivity [23]. Plants have evolved sophisticated mechanisms to perceive stress signals and initiate adaptive responses. Among these, phytohormones (Figure 1) play a pivotal role in orchestrating a wide range of physiological and molecular responses to abiotic stress [24]. Phytohormones, both classical (auxins, cytokinins, ethylene, GAs) and newer ones (brassinosteroids, jasmonates, strigolactones), play key roles in stress response and are promising targets for metabolic engineering [3,25]. These small signaling molecules act both locally and systemically to regulate gene expression, modulate metabolic pathways, and facilitate stress tolerance [26]. Phytohormones play a crucial role in protecting plants against biotic stress by modulating immune responses and coordinating defense signaling pathways against pathogens and herbivores [25].

### 3.1. Abscisic Acid (ABA)

**Figure 2:**
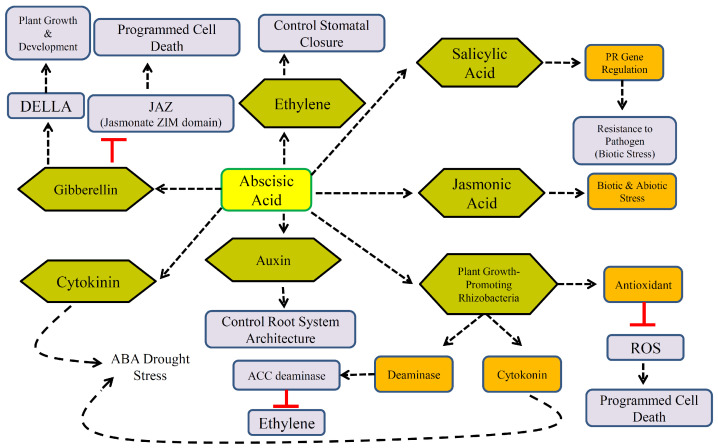
ABA-mediated hormonal response to biotic and abiotic stress in relation to other hormones.

ABA plays a pivotal role in plant responses to environmental challenges, earning its designation as the “stress hormone.” Its function is especially critical in response to abiotic stressors such as drought and salinity, where it serves as a central mediator of stress adaptation [27]. Under stressful environmental conditions, endogenous ABA levels increase swiftly, initiating a cascade of physiological responses. One of the earliest responses is the closure of stomata (Figure 2), which significantly reduces transpiration and conserves water. Concurrently, ABA modulates the expression of a suite of stress-responsive genes that underpin the plant’s physiological and biochemical adaptation.

The ABA signaling pathway consists of several key components. The PYR/PYL/RCAR family of receptors binds ABA, which then inhibits PP2C phosphatases. This inhibition lifts the suppression of SnRK2 kinases (Supplementary File), enabling them to phosphorylate and activate various transcription factors and stress response elements [28]. Through this mechanism, ABA stimulates the accumulation of osmoprotectants, increases the activity of antioxidant enzymes, and changes root system architecture, contributing to the plant’s stress tolerance and survival [29]. Chemically, ABA is a small sesquiterpene molecule with a non-planar structure and various functional groups that provide its activity and interaction potential [30]. Plants synthesize ABA de novo, especially during dehydration, and its levels drop when they are rehydrated. It is most abundant in roots and terminal buds. The biosynthesis starts from C15 carotenoid precursors such as xanthoxin and abscisic aldehyde [31]. Fundamental enzymatic steps involve converting β-carotene to intermediate products using gene-encoded enzymes that are upregulated in response to environmental stimuli [32].

The rate-limiting step in ABA biosynthesis is catalyzed by the enzyme 9-cis-epoxycarotenoid dioxygenase (NCED), which is quickly upregulated during drought and dehydration across many plant species [33]. Zeaxanthin epoxidase (ZEP), mainly found in the leaves, also plays a role in this biosynthetic pathway. While ABA can stimulate its breakdown through enzymes such as ABA 8′-hydroxylase, it does not promote its production [34]. Genes such as AAO3 (aldehyde oxidase 3) and MCSU (molybdenum cofactor sulfurase) are responsive to exogenous abscisic acid (ABA), suggesting the presence of intricate feedback regulatory mechanisms. ABA plays a central role in activating plant defense responses [35]. It contributes to cellular homeostasis by engaging common signaling pathways involved in mitigating stress-induced dehydration and osmotic imbalance [36].

Among environmental pollutants, heavy metals including cadmium (Cd), copper (Cu), lead (Pb), mercury (Hg), and chromium (Cr) cause serious abiotic stress for both plant and human health due to human activities. Cd is especially harmful, disrupting photosynthesis, growth, and stomatal regulation in plants [37]. ABA plays a role in Cd stress by affecting uptake and tolerance, with its effects often depending on the species. Both endogenous and exogenous ABA treatments have been shown to control stress-related gene expression, including NCED, glutathione peroxidase (GPX), and aquaporins [38]. It also boosts antioxidant responses and interacts with other stress signals, such as drought, to fine-tune defense mechanisms [39]. Copper (Cu) stress activates ABA-mediated signaling pathways, influencing genes related to oxidative stress response and stomatal regulation [40]. The co-application of protective agents such as silicon enhances ABA-induced stress tolerance by reducing metal uptake and strengthening plant defenses [41]. Drought, affecting over half of the world’s cultivable land, remains a major constraint to agricultural productivity. Under water-deficit conditions, plants accumulate ABA to stabilize membranes, regulate water balance, and adjust metabolic activity [17]. The exogenous application of ABA and signaling molecules including γ-aminobutyric acid (GABA) and salicylic acid (SA) can further boost drought resilience [42]. This response is primarily mediated by the core ABA signaling components: PYR/PYL/RCAR receptors, PP2C phosphatases, and SnRK2 kinases, which coordinate downstream dehydration responses [28].

Transcription factors also play an essential role in modulating ABA responses during drought. The bZIP family regulates numerous drought-related genes. In ramie, the BnbZIP3 gene is strongly induced by drought stress, enhancing ABA signaling [43] The ABA-dependent pathway of drought response involves ABRE (ABA-responsive element) motifs in gene promoters, while calcium signaling acts as a secondary messenger in both pathways, facilitating coordinated responses to multiple stress types including salinity and low temperature [44]. Water deficiency disrupts physiological functions such as photosynthesis, transpiration, and the biosynthesis of essential metabolites. Under these conditions, ABA levels are significantly elevated in stressed tissues compared to well-hydrated plants. While increased ABA aids in stress adaptation, excessively high concentrations may suppress shoot growth. However, root development is often maintained or even enhanced through ABA-mediated crosstalk with other hormones, such as ethylene [45]. ABA promotes the accumulation of protective molecules such as late embryogenesis abundant (LEA) proteins and compatible solutes that support osmotic balance [46].

ABA transport is another crucial aspect of its functional role. Transporter proteins such as AtABCG25 facilitate the export of ABA from cells (vascular tissues, including phloem companion cells), mediating its systemic effects across plant tissues [47]. Overexpression of AtABCG25 in experimental models has been associated with enhanced drought tolerance due to reduced water loss, without impairing growth, highlighting its potential for crop improvement [48]. Ultraviolet (UV) radiation, especially UV-B (280–315 nm), adds a different type of abiotic stress. While UV-C is fully absorbed by the ozone layer, UV-B can partially penetrate and harm plant cells by producing ROS [49]. ABA can help lessen the impact of UV-B by causing stomatal closure, lowering ethylene levels, and boosting overall stress resilience [50]. Research in maize shows that UV-B exposure raises ABA levels, which increases signaling molecules such as hydrogen peroxide (H_2_O_2_) and nitric oxide (NO). These molecules are crucial for defense responses [51].

In addition to helping plants with stress, ABA also plays a key role in developmental processes such as seed dormancy, germination, and seedling growth. Several *Arabidopsis* genes, including *abi1*, *abi2*, *abi3*, *abi4*, and *abi5*, have been identified as important in these processes [52]. The *abi1* and *abi2* genes act as negative regulators, especially affecting germination and stomatal function [53] Meanwhile, *abi5* impacts seedling growth and ABA sensitivity [54]. Members of the SnRK2 family, e.g., SnRK2.2 and SnRK2.3, promote ABA-induced seed dormancy and stop seedling growth by activating gene networks containing ABA responsiveness elements [55] These kinases likely work by adding phosphate groups to ABA-responsive transcription factors (ABFs), which improves their ability to bind DNA and boosts the transcription of specific stress-response genes [56].

### 3.2. Salicylic Acid (SA)

SA, known for its role in plant defense against biotic stress and pathogens, also plays an important part in managing abiotic stress responses [57]. It helps regulate the antioxidant defense system, maintaining cellular balance under stress conditions [58]. SA has been found to reduce the negative effects of salinity, heavy metals, and heat by triggering the expression of stress-related genes and increasing the activity of antioxidant enzymes such as catalase, peroxidase, and superoxide dismutase [58].

One study highlighted SA’s effect on salinity tolerance in two wheat cultivars (SST806 and PAN3497) exposed to 100 and 200 mM NaCl stress. Salt stress (rinsing seeds in 0.1% sodium hypochlorite) severely impacted germination, growth, and yield traits. When 0.5 mM SA was applied by spraying, it helped overcome these issues by improving shoot and root biomass, yield attributes, and grain protein content, especially in SST806. The application also enhanced the uptake of minerals such as Na, Mn, Zn, Fe, and Cu, suggesting better nutrient flows [59]. In rice varieties (BRRI dhan66 and BRRI dhan75), a treatment combining 0.75 mM SA and 1 mM proline under drought stress provided better results than individual treatments. This combination boosted antioxidant activity, increased osmoprotectant levels, and improved photosynthesis and water content, leading to greater biomass and yield [60].

In *Brassica napus*, applying SA helped reduce the impact of drought and salinity stresses. Under stress conditions, the plants showed decreased leaf area, less chlorophyll content, and reduced gas exchange, along with increased oxidative stress and structural damage. The use of SA improved physiological and growth factors, lowered oxidative stress markers such as MDA, and helped maintain leaf structure, highlighting its potential to improve plant resilience [61]. A similar positive effect was seen in *Camelina sativa* under salt stress. The combination of SA and 24-epibrassinolide (EbR) enhanced relative water content, as well as fresh and dry weights, and increased pigment levels. This combined treatment significantly improved salt stress tolerance, especially in the PI-650142 genotype [62].

Drought tolerance is associated with changes in gene expression such as *LTI29* and *LTI30*, encoding dehydrins, which are upregulated under drought to help maintain cellular stability [63]. Endogenous SA levels also rise under drought, promoting the expression of pathogenesis-related (PR) genes such as *PR1* and *PR2* [57]. SA enhances the expression of antioxidant genes, including glutathione S-transferases, ascorbate peroxidase (APX), and 2-cysteine peroxiredoxin, helping reduce oxidative damage [64]. *Arabidopsis* mutants with elevated SA levels (*adr1*, *siz1*, *cpr5*, *acd6*, *myb96-1d*) show enhanced drought tolerance due to SA-dependent gene regulation. In potatoes under moderate water stress, SA application improved tolerance by boosting antioxidant defense, reducing ROS, maintaining membrane integrity, and enhancing bioactive compound synthesis [65].

SA also plays a pivotal role in the cold stress response. Low concentrations (0.1–0.5 mM) improve cold tolerance in crops such as maize, rice, tomato, and pepper by promoting antioxidant defenses and inducing genes such as Alternative Oxidase (AOX) [66]. However, excessive or prolonged SA application can be harmful, causing oxidative damage and reduced cold tolerance [67]. *Arabidopsis* mutants that overaccumulate SA (*siz1*) are sensitive to freezing, while SA-deficient mutants (*nahG*, *eds5*) show increased cold resilience. Cold-responsive transcription factors such as CAMTA3/AtSR1 regulate the balance between stress response and defense, promoting CBF2/DREB1C while repressing EDS1, reflecting the complex interaction between SA signaling and cold stress pathways [68].

### 3.3. Jasmonic Acid (JA)

JA and its derivatives, jasmonates, are lipid-based phytohormones involved in mediating plant responses to both biotic and abiotic stresses. Under abiotic stress, JA regulates root growth, stomatal conductance, and antioxidant metabolism [69]. Its biosynthesis and signaling are activated under stress, resulting in the accumulation of protective compounds including proline and flavonoids [70]. JA often operates through intricate cross-talk with other hormones, including abscisic acid and salicylic acid, to fine-tune stress responses [71]. During drought stress, JA’s precursor oxophytodienoic acid (OPDA) accumulates and promotes stomatal closure, conserving water [72]. Lipoxygenase (LOX) genes, particularly *LOX6*, enhance OPDA synthesis, supporting drought tolerance even in the absence of ABA. In rice, OsbHLH148 activates drought-responsive genes through interactions with OsJAZ1, a repressor of jasmonic acid (JA) signaling, and OsCOI1, an essential JA receptor, thereby linking JA signaling to drought stress responses [73]. Overexpression of JA-regulated genes such as *CMLOX10* in melon and *RhHB1/RhLOX4* in rose enhances drought resilience [74]. NAC transcription factors such as VvNAC17 (NAC transcription factor 17 from *Vitis vinifera*) and VvNAC26 (NAC transcription factor 26 from *Vitis vinifera)* further modulate JA-related stress responses [75]. In *Arabidopsis*, ICE1/2 transcription factors activate the CBF pathway, which is crucial for cold tolerance [76]. JAZ proteins normally repress this pathway, but, under cold stress, JA degrades JAZ repressors via the COI1–JAZ complex, enabling ICE–CBF activation [77]. Cold-induced upregulation of genes including *AOC*, *AOS1/2*, *LOX2*, *COI1a*, and *bHLH148* boosts JA signaling [70]. In apples, MdMYC2 enhances cold tolerance, counteracted by overexpressed MdJAZ1/4 [78], and MdBBX37 promotes cold resistance but is suppressed by MdJAZ1/2 [79,80]. JA-responsive MYC2-like transcriptional regulatory element induces secondary metabolites like artemisinin and glycine betaine, aiding cold adaptation [81]. Exogenous methyl jasmonate (MeJA) enhances cold tolerance in pepper and lemon, mitigating chilling injury [82]. JA supports salt stress adaptation via *LOX3*, *MYC2*, and *GTR1* [83]. In rice, OsbHLH062 and OsJAZ9 maintain ion homeostasis. OsbHLH062, when relieved of repression by OsJAZ9 (upon JA signaling activation), can induce genes such as ion transporters or antiporters (e.g., HKT, NHX, or SOS family members), contributing to ion homeostasis and reduced Na^+^ toxicity [84]. Moreover, GaJAZ1 (JAZ family protein) enhances salt tolerance in cotton by modulating JA-responsive gene expression that controls both ionic and oxidative stress pathways [85]. JA also enhances thermotolerance by improving antioxidant defenses and osmotic regulation [86]. However, in rice, JA-induced stomatal closure may impair heat dissipation [87].

JA mitigates heavy metal toxicity by limiting metal uptake and boosting antioxidant systems. MeJA application reduces cadmium accumulation by downregulating AtIRT1, AtHMA2, and AtHMA4 [88]. JA also alleviates nickel, Cu, and lead stress in several crops, often synergistically with SA and proline [89].

### 3.4. Ethylene

Ethylene is a gaseous plant hormone integral to development and stress responses [90]. Its biosynthesis often increases under abiotic stress, influencing plant adaptation in a context-, concentration-, and timing-dependent manner [91]. Ethylene interacts with other hormones such as ABA and JA to regulate processes including leaf senescence, stomatal behavior, and root development [92]. Transcription factors, including EIN3 and ethylene response factors, mediate ethylene signaling under stress [93]. Ethylene biosynthesis begins with methionine and involves three enzymes, SAM synthetase, ACC synthase (ACS), and ACC oxidase (ACO), with ACO catalyzing the final step [94]. Genes such as *PhACO1*, *PhACO3*, and *PhACO4* regulate ethylene levels, as shown by CRISPR/Cas9 studies that altered ethylene production and impacted flower longevity and seed germination [95]. Ethylene’s impact on stress tolerance differs among species and gene expression. For example, ETR1 is increased in salt-stressed cotton [96] but decreased in *Arabidopsis* [97]. Ethylene signaling controls antioxidant defenses and osmotic balance [98]. Compounds such as ethephon improve tolerance by adjusting ROS levels and shifting methionine toward glutathione production [90].

### 3.5. Auxins

Auxins, especially indole-3-acetic acid (IAA), play a key role in plant growth. They influence cell elongation, division, and differentiation [99]. When plants face abiotic stress, auxin production, movement, and signaling change, which affects root structure and stress responses. Auxin transporters, including PIN proteins, and signaling components, such as ARFs and AUX/IAAs, control gene expression related to stress tolerance [100]. Salt stress increases the expression of auxin biosynthesis genes such as *NIT1*, *NIT2*, and *YUC4*, which helps improve tolerance [99]. Genes from the IAOx pathway, including CYP79B2 and CYP79B3, show increased expression under mild salinity, likely to enhance auxin biosynthesis and indole glucosinolate production, thereby promoting root adaptation, ion balance, and oxidative stress mitigation [99]. Maintaining auxin balance is vital; losing GH3 enzymes increases salt tolerance [101]. Salt stress interferes with auxin transport by reducing PIN1, PIN3, and PIN7 expression [102]. Meanwhile, PIN2, AUX1, and LAX3 help bend roots away from salty areas [103]. Mutations in auxin receptors TIR1, AFB2, and AFB3 cause hypersensitivity to salt. However, overexpressing these receptors improves tolerance [104]. Aux/IAA proteins, including IAA17, and ARFs such as OsARF11 and IbMP/ARF5 are influenced by salt stress and miRNAs.

During drought, auxin coordinates with ABA to regulate stress responses. Auxin biosynthesis via YUC genes enhances drought tolerance by activating ABA-responsive genes and ROS detoxification [105]. Auxin transporters (PIN, LAX) are upregulated under drought [102], and signaling components such as OsIAA18, IbARF5, and SlARF4 are crucial regulators of drought responses [106]. Under cold stress, auxin biosynthesis is induced (e.g., increased YUC expression), while auxin transport is disrupted by the mislocalization of PIN2 and PIN3 [107]. Auxin and its analogs enhance cold tolerance by upregulating genes involved in the biosynthesis of cryoprotectants such as proline and soluble sugars, as well as by modulating ROS-scavenging enzyme activities and maintaining membrane fluidity [108]. Overexpression of SgGH3.1 and CsARF5 improves chilling tolerance [109], while IAA14 mutants display increased cold sensitivity [110]. Cold acclimation involves sensors such as calcium channels and COLD1 and activation of the CBF/DREB1 pathway [111], where auxin signaling integrates into broader stress regulatory networks.

### 3.6. Cytokinins

Cytokinins play complex and multifaceted roles in enhancing plant tolerance to drought and salinity, two major abiotic stresses affecting global crop productivity [112]. Under water-limiting conditions, plants often downregulate cytokinin levels through changes in metabolism, receptor expression, or the activation of negative regulators including AHP6 and ARR5 [113]. Strategic modulation of cytokinin signaling improves stress tolerance through five primary mechanisms: preserving photosynthesis, boosting antioxidant defenses, enhancing water regulation, influencing plant growth, and interacting with stress-related hormones [114]. Elevated cytokinin levels can enhance photosynthetic performance by upregulating genes associated with chlorophyll biosynthesis and CO_2_ assimilation; in contrast, excessive cytokinin degradation can impair photosynthesis [115]. Cytokinins also support antioxidant systems by increasing antioxidant and flavonoid production, mitigating ROS damage [112]. Regarding water balance, lower cytokinin levels promote root development and water retention, while reducing transpiration and stomatal conductance [116]. In terms of growth, modifying cytokinin levels, particularly via root-specific CKX overexpression, improves root architecture, biomass, and recovery post-drought without hindering shoot development [117].

### 3.7. Gibberellins (GAs)

The discovery of bioactive gibberellins (GAs) originated from studies on *Gibberella fujikuroi* infection in rice. Since then, over 100 types of GAs have been identified, though only a few, such as GA_1_, GA_3_, and GA_4_, are biologically active, depending on the species [118]. GAs play crucial roles in multiple plant developmental processes, including germination, seedling growth, stem and root elongation, leaf and flower formation, fruit development, and pollination [119]. DELLA proteins, key repressors of GA signaling, accumulate under stress conditions and modulate gene expression to enhance stress tolerance. Their interactions with other hormone pathways reveal a complex hormonal crosstalk that fine-tunes plant responses to environmental challenges [120] [121]. Interactions between GAs and ABA are particularly important in seed dormancy and germination under adverse conditions [119]. A study investigating the effects of GA_3_ seed priming on salt tolerance in two wheat cultivars (MH-97, salt-intolerant; Inqlab-91, salt-tolerant) under 15 dS m^−1^ NaCl salinity found that 150 mg L^−1^ GA_3_ was especially effective in improving grain yield, particularly in the salt-intolerant cultivar. GA_3_ priming reduced Na^+^ accumulation and increased Ca^2+^ and K^+^ levels in roots under salt stress. While GA_3_ had little consistent effect on gas exchange and auxin levels, it reduced ABA levels in the salt-intolerant cultivar and enhanced SA levels in both cultivars. GA_3_ priming lowered levels of polyamines such as putrescine and spermidine, thereby reducing stress. Polyamines are small aliphatic amines involved in stabilizing membranes, scavenging reactive oxygen species, and regulating gene expression during stress responses; however, their excessive accumulation under certain conditions may exacerbate stress damage [121,122]. The rice cytochrome P450 gene *OsCYP71D8L*, a member of the CYP71 clan, regulates plant growth and abiotic stress responses by modulating GA and cytokinin homeostasis. Gain-of-function mutants and overexpression lines (CYP71D8L-OE) showed dwarfism, reduced panicle length, and fewer grains per panicle, along with lower GA levels. These growth defects were rescued by GA_3_ or a cytokinin biosynthesis inhibitor (lovastatin) and worsened by synthetic cytokinin (6-BA), indicating hormonal imbalance [123]. A study demonstrates that *VvGA2ox7*, a gene from a grape encoding a GA-deactivating enzyme, enhances salt stress tolerance when overexpressed in *Arabidopsis thaliana*. The VvGA2ox7 protein contains typical 2-oxoglutarate-dependent dioxygenase domains and is localized in the nucleus and cytoplasm. Overexpression led to reduced GA_3_ levels, increased antioxidant enzyme activities, upregulation of salt stress-responsive genes, higher proline, ABA, and chlorophyll contents, and decreased membrane damage indicators such as malondialdehyde and relative conductivity [124].

### 3.8. Brassinosteroids (BRs)

BRs are steroidal hormones that regulate cell expansion, vascular differentiation, and stress responses. BR signaling enhances tolerance to various abiotic stresses by modulating antioxidant systems, heat shock proteins, and stress-responsive transcription factors. BZR1 and BES1 are key BR-responsive transcription factors that integrate environmental signals and hormonal cues to mediate stress adaptation [125].

BRs, particularly 24-epibrassinolide (EBR), enhance thermotolerance and salt tolerance in *Arabidopsis*, largely through interactions with other plant hormones [126]. BRs have been extensively shown to enhance plant tolerance to both high- and low-temperature stresses by modulating the antioxidant defense system. Under high-temperature conditions, BR treatments improved stress tolerance in various crops (rice, mung bean, tomato) by increasing antioxidant enzyme activities (SOD, POD, CAT, APX), reducing lipid peroxidation and ion leakage and enhancing membrane stability and water potential [127]. During low-temperature stress, BRs (such as 24-epiBL and 28-homoBL) alleviated chilling injuries by boosting antioxidant enzyme activity, increasing proline and phenolic contents, and maintaining membrane integrity [127]. BRs also protect photosynthetic efficiency and regulate osmoregulation, contributing to better stress recovery [128].

## 4. Molecular Mechanisms of Hormonal Crosstalk Under Abiotic Stress

Plants respond to abiotic stresses such as drought, salinity, and temperature extremes through complex signaling networks, prominently regulated by hormonal crosstalk. Under drought and salinity stress, ABA signaling becomes dominant. ABA biosynthesis is upregulated via NCED3 [129], while its perception involves PYL4/5/8 receptors that inhibit the negative regulators ABI1 and ABI2, thereby activating SnRK2 kinases (SnRK2.2/2.3/2.6). These kinases phosphorylate transcription factors such as AREB1 and ABF2, leading to the induction of stress-responsive genes [130]. ABA exhibits antagonism with salicylic acid (SA); ABA-activated SnRK2.6 suppresses NPR1, a central regulator of SA signaling, thereby prioritizing drought tolerance over pathogen defense [131]. SA signaling, primarily mediated through ICS1, NPR1, and TGA transcription factors [132], modulates oxidative stress responses by upregulating PR genes (*PR1*, *PR2*, *PR5*) [133]. Under combined abiotic–biotic stress, EDS1-PAD4-NPR1 complexes integrate signals to enhance basal defense while mediating crosstalk with JA and ABA pathways [134]. NPR1 can also repress JA-responsive genes through transcriptional interference [135]. In the JA pathway, COI1 forms a receptor complex with JAZ repressors, which are degraded upon JA accumulation, releasing transcription factors including MYC2 [136]. MYC2 also acts as a node for ABA-JA crosstalk, where it integrates JA responses with ABA-mediated drought tolerance. JA and ET often act synergistically; EIN3, a key ET signaling protein, cooperates with MYC2 to co-regulate stress and defense-related genes [137].

Ethylene signaling is initiated by ETR1 and transduced via CTR1 and EIN2, leading to EIN3/ERF1 activation [138]. Ethylene-ABA antagonism is evident at the level of SnRK2.6 and EIN3, where ethylene suppresses ABA-induced stomatal closure, balancing stress signaling and growth [139]. Auxin, through TAA1, YUC6, AUX1, and PIN transporters, modulates root architecture under stress [140]. Stress-induced ABA inhibits auxin transport (PIN1, PIN3) and signaling (ARF7, ARF19) to reprogram growth [141]. Simultaneously, CK signaling (AHK2/3, ARR1/10), which promotes cell division, is suppressed by ABA to reduce growth during stress [13]. Type-A ARR5 is induced under drought via ABA-SnRK2 signaling, indicating tight feedback regulation [142]. GA signaling, governed by GID1, is negatively regulated by DELLA proteins (GAI, RGA, RGL2) under stress, integrating with ABA to restrain growth [143]. ABA enhances DELLA stability, promoting stress resistance over growth [144].

BR signaling, involving BRI1, BAK1, and transcription factors BZR1/BES1, promotes growth under abiotic stress [145]. BR interacts positively with ABA to maintain membrane integrity and photosynthetic efficiency, particularly through BZR1-mediated antioxidant gene regulation [146].

**Figure 3:**
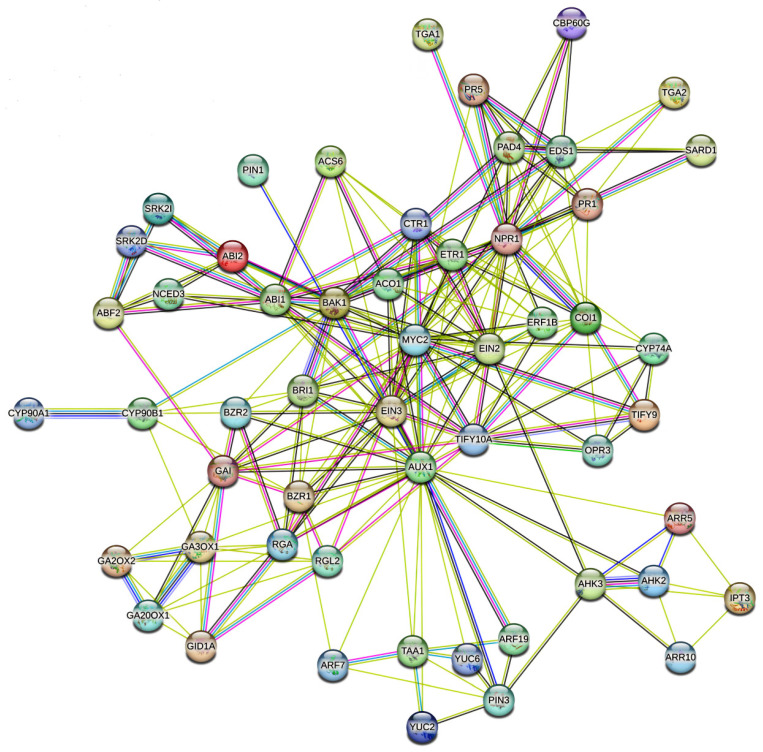
STRING networking of hormonal crosstalk dynamics under stress. The interaction was created using the STRING database (https://string-db.org/ (accessed on 12 February 2025) with respect to the *Arabidopsis* database (network nodes represent proteins, colored nodes indicates query proteins and the first shell of interactors, white nodes indicate the second shell of interactors, empty nodes indicate proteins of unknown 3D structure, and filled nodes indicate 3D structure proteins of known or predicted structures), which enables the visualization of protein–protein interaction (PPI) networks containing 54 node connected by 215 edges, having a PPI enrichment of *p* < 1.0 × 10^−16^. The average node degree is 7.96 and the average local clustering co-efficient is 0.677. The interactions were mapped using multiple data sources, curated databases, and co-expression analyses. A high confidence score threshold was applied to ensure reliability, and the resulting network reflects distinct hormonal interactions. Colored lines represent various types of evidence supporting interactions. Green shows gene neighborhood, red indicates gene fusion, and blue reflects gene co-occurrence across genomes. Pink lines represent experimental evidence, light blue denotes curated database information, black shows co-expression, yellow indicates text mining, and lilac suggests protein homology. The thickness of the lines corresponds to confidence levels, with thicker lines indicating stronger evidence.

This hormonal crosstalk ensures the dynamic reallocation of resources under stress. The STRING (Search Tool for the Retrieval of Interacting Genes/Proteins) database plays a pivotal role in decoding the molecular mechanisms of hormonal crosstalk under abiotic stress by systematically integrating known and predicted protein–protein interactions. In the context of plant stress biology, hormonal signaling STRING (Figure 3) analysis has 54 nodes interconnected with 215 edges, with an average node degree of 7.96 at PPI enrichment *p*-value < 1.0 × 10^−16^. Core interaction nodes such as SnRK2s, MYC2, NPR1, EIN3, and BZR1 show the important roles of transcription networks in adjusting hormone signaling. Understanding these interactions offers valuable insights for engineering crops that are more resilient to environmental stressors.

The STRING-based analysis of hormonal crosstalk genes under abiotic stress shows a complex but coordinated regulatory network in plants. Key molecular functions such as binding activity, transcription regulation, and signal transduction are noticeably enriched. A large portion of the proteins analyzed are involved in hormone perception and signal relay. This includes receptor kinases such as BRI1 (brassinosteroids), AHK2/3 (cytokinins), and ETR1 (ethylene), which are critical for early signaling events. Transcription factors including MYC2 (jasmonate), EIN3 (ethylene), ABF2 (ABA), and ARFs (auxin) were identified as key regulators. This suggests a broad reprogramming of transcription in response to abiotic stress.

The enrichment of genes with oxidoreductase and dioxygenase activity, including GA3OX1, NCED3, and YUC family members, highlights the active adjustment of hormone biosynthesis pathways. Auxin transporters such as AUX1 and PINs further stress the role of hormone distribution in adapting to stress. These findings highlight the importance of protein–protein interactions, kinase signaling, and transcriptional control in managing the hormonal interactions needed for resilience to abiotic stress. Overall, this analysis provides a systems-level view of how plants combine multiple hormone signals through key molecular functions to adjust their physiological responses to challenging environmental conditions.

**Figure 4:**
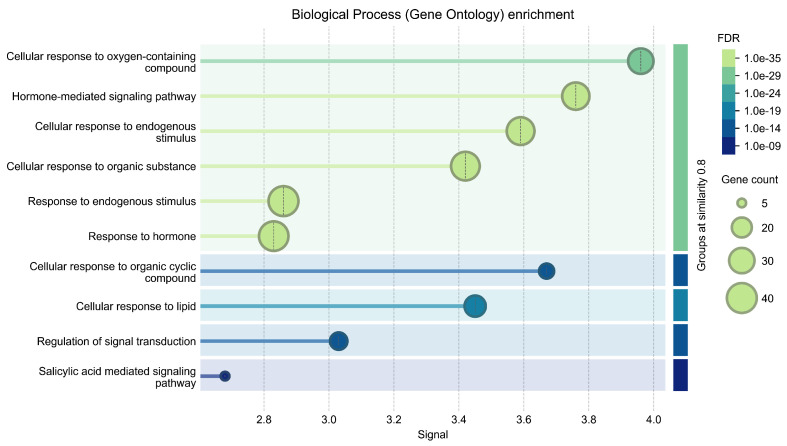
Enrichment gene ontology of major biological processes detected through STRING analysis of the hormonal crosstalk dynamic at similarity ≥ 0.8.

The STRING-based Gene Ontology enrichment analysis of hormone-responsive genes under abiotic stress highlights several interconnected biological processes that reflect the complex molecular mechanisms of hormonal crosstalk in plants. Genes involved in key stress-related hormones, such as ABA (e.g., *PYL4*, *ABI1*, *SnRK2s*), JA (*COI1*, *JAZs*, *MYC2*), SA (*NPR1*, *ICS1*), ethylene (*ETR1*, *EIN2*, *EIN3*), auxin (*ARFs*, *PINs*), and BRs (*BRI1*, *BZR1*) participate in pathways related to cellular responses to oxygen-containing compounds, endogenous stimuli, and organic substances. These genes modulate stress tolerance by regulating hormone-mediated signaling, redox homeostasis, osmolyte accumulation, and membrane stability. The enrichment of processes including the “salicylic acid-mediated signaling pathway,” “cellular response to lipid,” and “regulation of signal transduction” suggests that these genes integrate diverse signals, including ROS and lipid-derived messengers, to fine-tune adaptive responses. Notably, the salicylic acid pathway genes (NPR1, ICS1) play pivotal roles in managing oxidative stress and priming plants for combined biotic–abiotic stress resistance. Altogether, this enrichment analysis emphasizes the coordinated action of hormone signaling networks in regulating plant physiology and resilience under abiotic stress through tightly controlled gene expression and signal transduction mechanisms (Figure 4).

The k-means clustering (Supplementary File) and KEGG pathway (Table 1) enrichment analyses collectively provide insight into the molecular mechanisms underlying hormonal crosstalk in plants under abiotic stress. Clustering classified hormone-responsive genes into six functionally distinct groups based on shared expression patterns and biological roles. Cluster 1 was strongly aligned with the MAPK signaling pathway—plant, incorporating key stress and hormone-responsive genes such as *MYC2*, *EIN2*, *COI1*, *ACS6*, and *ETR1*, which also appeared significantly enriched in the KEGG term ath04016 (MAPK signaling), reinforcing their central role in transducing abiotic stress signals. Cluster 2 grouped genes involved in GA signaling and diterpenoid biosynthesis, including GA3OX1, GA20OX1, GA2OX2, and DELLA repressors, which matched well with ath00904 and ath00905 (Diterpenoid and BR biosynthesis), indicating coordination between growth regulation and stress response. Cluster 3 was enriched in hypersensitive response and systemic acquired resistance genes such as *NPR1*, *PR1*, and *TGA2*, and it overlapped with terms related to salicylic acid signaling in the GO analysis, highlighting their immune-regulatory roles. Cluster 4 represented auxin-related genes (e.g., *PINs*, *YUCs*, *ARFs*), mapping to ath00380 (Tryptophan metabolism), consistent with auxin biosynthesis pathways. Cluster 5 included ABA pathway regulators such as ABI1, ABI2, and NCED3, which are critical in drought response, and connected to ath04075 (plant hormone signal transduction). Lastly, Cluster 6 included cytokinin signaling components such as ARR5, AHK2, and AHK3, also enriching ath04075. Thus, a strong correlation exists between the k-means cluster identities and KEGG pathway annotations, confirming that distinct hormone signaling modules operate in parallel yet interlinked networks to orchestrate complex adaptive responses under abiotic stress conditions.

## 5. Crosstalk Dynamics: Specific Hormonal Interactions

Plant hormones, or phytohormones, act as signaling molecules that control various developmental processes and stress responses. Instead of working alone, these hormones often interact through complex signaling networks, a process known as hormonal crosstalk [147]. Crosstalk dynamics refers to how different hormonal pathways coordinate in space and time. This coordination allows plants to combine various internal and environmental signals and make precise physiological responses [148]. Certain hormonal interactions, like those between ABA and ethylene, auxin, and cytokinin, or SA and JA, involve changes at multiple levels. These include biosynthesis, receptor signaling, transcriptional regulation, and protein stability [149]. These interactions can be either helpful or harmful, depending on the developmental context or type of stress faced [134]. Understanding how these interactions work is essential for clarifying the hormonal networks that control plant plasticity. It also provides valuable opportunities for manipulating hormone signaling pathways to boost stress tolerance, growth, and productivity in crops. Studying specific hormonal crosstalk dynamics is an important area in plant molecular biology and agricultural biotechnology.

### 5.1. ABA–Ethylene Crosstalk

ABA and ethylene are key plant hormones that play important roles in regulating plant growth and responses to stress. The interaction between ABA and ethylene is complex and can affect plant behavior in various and often opposing ways.

**Figure 5:**
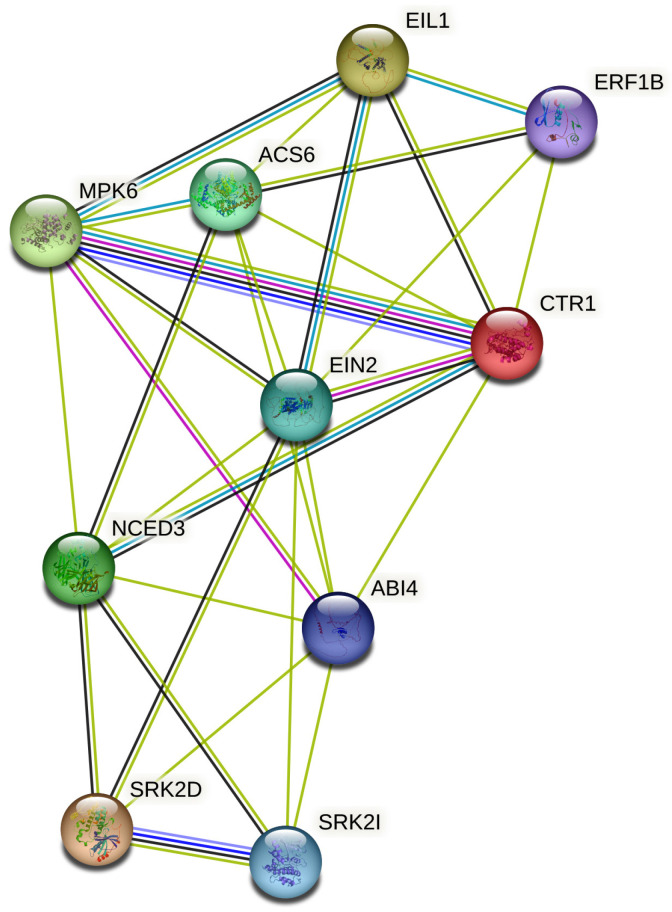
STRING analysis detecting protein–protein interaction dynamics of ABA–ethylene crosstalk. The analysis was performed by inputting a curated list of ABA- and ethylene-responsive genes into the STRING database with reference to *Arabidopsis* database. This analysis mapped the interactions between major signaling components and downstream effectors, emphasizing both direct and indirect associations within and across the two hormonal pathways. The interaction detected 10 nodes linked by 30 edges. Network nodes represent proteins, colored nodes indicate query proteins and the first shell of interactors, white nodes indicate the second shell of interactors, empty nodes indicate proteins of unknown 3D structure, and filled nodes indicate 3D structure proteins of known or predicted structures. The average node degree is 6 and the average local clustering coefficient is 0.83. The resulting protein–protein interaction (PPI) network (*p*-value: <0.83) revealed functional modules and hub proteins involved in stress responses. Colored lines represent various types of evidence supporting interactions. Green shows gene neighborhood, red indicates gene fusion, and blue reflects gene co-occurrence across genomes. Pink lines represent experimental evidence, light blue denotes curated database information, black shows co-expression, yellow indicates text mining, and lilac suggests protein homology. The thickness of the lines corresponds to confidence levels, with thicker lines indicating stronger evidence.

One well-known outcome of ABA-ethylene interaction occurs during seed germination. ABA promotes dormancy and hinders germination by activating genes that keep seeds inactive. Ethylene, on the other hand, encourages seed germination by counteracting ABA signaling. This counteraction happens on multiple levels, including biosynthesis, perception, and downstream signaling [150]. Ethylene can lower the expression of ABA biosynthetic genes, e.g., *NCED*, resulting in reduced ABA levels in seeds [139]. Components of ethylene signaling, such as EIN3 and EIL1, negatively affect ABA-responsive transcription factors, e.g., ABI3 and ABI5, which are crucial for maintaining dormancy [151]. By destabilizing or suppressing these ABA regulators, ethylene encourages germination, especially in conditions that support growth. ABI4 serves as a key regulator by inhibiting genes related to ethylene production, such as ACS6. This support for ABA responses includes seed dormancy and stress tolerance [152]. Conversely, elements of the ethylene signaling pathway, including EIN2, EIL1, and ERF1, can alter ABA responses, often in opposing ways during seed germination and root growth. The kinase MPK6 plays a role in both pathways by activating ACS genes and combining ABA signals in stress situations. Negative regulators such as CTR1 reduce ethylene signaling, and ABA’s influence on them adds another layer of complexity. ABA production is strictly controlled by NCED3, which can be affected by ethylene in response to certain environmental signals, showing feedback regulation [153]. ABA signaling kinases SnRK2.2 and SnRK2.3 also adjust the expression of ethylene-responsive genes, creating a coordinated hormonal response [55] (Figure 5).

The opposing relationship between ABA and ethylene is also evident in regulating stomatal movement, which is vital for conserving water during drought. ABA encourages stomatal closure to reduce water loss, a process facilitated by secondary messengers such as ROS and calcium ions [154]. Ethylene can disrupt ABA-induced stomatal closure by blocking the ROS signaling pathway in guard cells, which decreases ABA sensitivity [155]. However, ethylene’s effect is not always opposing; in some stress situations, it may enhance ABA’s influence on guard cells, indicating a complex and context-specific interaction [92,155]. Ethylene’s dual role in modifying ABA responses highlights the flexible nature of their interaction, which is influenced by developmental stage, environmental signals, and tissue type.

Regarding their opposing roles in seed germination and stomatal closure, ethylene often works together with ABA during leaf aging and dropping [139]. During aging or stress-induced senescence, ABA boosts ethylene production by increasing the expression of genes such as ACS (ACC synthase), which raises ethylene levels. This ethylene then promotes the expression of genes linked to senescence, speeding up the breakdown of cellular components and aiding nutrient remobilization [156]. The ABA–ethylene cooperation in senescence shows a coordinated approach to conserving energy and reallocating resources in tough conditions. This teamwork ensures that plants smoothly transition into energy-saving mode during times of limited water or nutrients.

The ABA–ethylene interaction also plays a role in how plants respond to abiotic stress. During drought or salinity stress, ABA levels jump quickly, triggering various protective actions such as stomatal closure, accumulating osmolytes, and expressing stress-related genes [154]. In the case of biotic stress, the ABA–ethylene interaction becomes even more complicated. Ethylene significantly regulates defense responses, especially against necrotrophic pathogens, often working alongside JA [157]. Generally, ABA suppresses the defense pathways driven by ethylene and JA, which can make plants more vulnerable to pathogens [158]. This suppression might be beneficial in abiotic stress situations, where ABA-driven stress resistance takes priority over defense. Thus, the interaction between ABA and ethylene represents a complex and responsive regulatory system that supports many important physiological processes in plants. This relationship can be either opposing or collaborative, depending on the specific developmental stage, tissue type, and environmental factors. Their mutual influence on each other’s production, perception, and signaling pathways allows ABA and ethylene to finely tune plant responses to a wide range of stimuli.

### 5.2. ABA–JA and JA–SA Interactions

ABA, JA, and SA are three important phytohormones that play crucial roles in how plants respond to various environmental stresses. These hormones do not work alone; they interact in complex networks to adjust plant functions [159]. The communication between ABA and JA, as well as between JA and SA, forms important regulatory mechanisms that balance growth and defense in plants [71]. Understanding these hormonal interactions is key to figuring out how plants manage multiple signals to survive in tough conditions.

**Figure 6:**
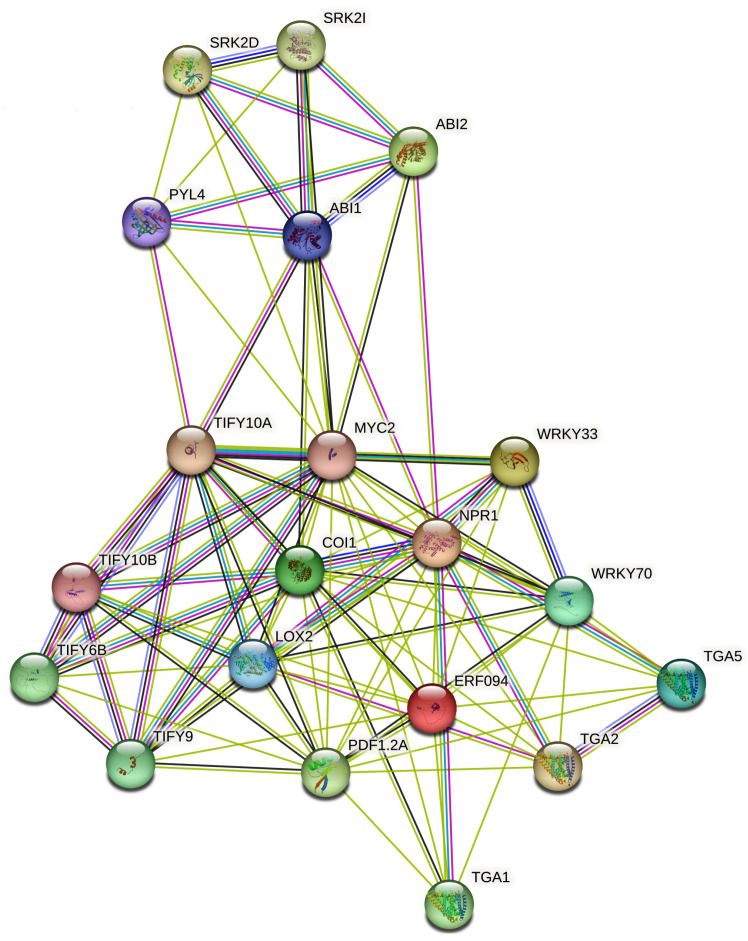
STRING analysis detecting the protein–protein interaction dynamics of ABA, JA, and SA, mapped with respect to the *Arabidopsis* database containing 20 nodes connected by 96 edges having a PPI enrichment of *p* < 1.0 × 10^−16^ and an average node degree of 9.8. Network nodes represent proteins, colored nodes indicate query proteins and the first shell of interactors, white nodes indicate the second shell of interactors, empty nodes indicate proteins of unknown 3D structure, and filled nodes indicate 3D structure proteins of known or predicted structures. Colored lines represent various types of evidence supporting interactions. Green shows gene neighborhood, red indicates gene fusion, and blue reflects gene co-occurrence across genomes. Pink lines represent experimental evidence, light blue denotes curated database information, black shows co-expression, yellow indicates text mining, and lilac suggests protein homology. The thickness of the lines corresponds to confidence levels, with thicker lines indicating stronger evidence.

The interaction between ABA-JA and JA-SA signaling pathways in plants involves a tightly controlled network of genes that manage responses to abiotic and biotic stresses (Figure 6). In the ABA–JA module, the transcription factor MYC2 plays a central role by connecting signals from both hormones [160]. JAZ repressors (JAZ1, JAZ2, JAZ3, JAZ10) negatively regulate MYC2 and are marked for destruction by the COI1 receptor when JA levels rise [161]. Components of ABA signaling, such as ABI1, ABI2, SnRK2.2, SnRK2.3, and SnRK2.6 (OST1), influence MYC2’s activity [162]. Additionally, ABF2 (AREB1), a bZIP transcription factor, also supports ABA-responsive gene expression [163]. ABA receptors including PYL4 and negative regulators such as PP2CA (AHG3) fine-tune the pathway by controlling SnRK2 activity [164].

In the JA–SA interaction module, SA signaling through NPR1 counteracts JA responses by repressing key genes, e.g., *ORA59*, which is necessary for JA/ethylene-responsive gene expression (e.g., PDF1.2) [165]. SA-inducible transcription factors such as WRKY70 and TGA1/2/5 promote the expression of *PR1*, a key SA-responsive gene [166], while downregulating JA biosynthetic genes such as *LOX2* and *AOS* [167]. EDS1 and PAD4 enhance SA signaling and further suppress JA-related defenses [57]. This complex hormonal interaction ensures that plants respond appropriately according to environmental cues. ABA–JA pathways often work together under abiotic stress, while JA–SA pathways tend to act against each other during pathogen defense.

### 5.3. ABA–Auxin Crosstalk

The interaction between ABA and auxin is a key part of hormonal communication that influences various plant development and stress response processes. The relationship between these two hormones changes based on different contexts. It involves complex signaling networks that manage physiological responses to developmental signals and environmental changes. At the biosynthetic and transport stages, ABA and auxin affect each other’s balance. ABA regulates the expression of auxin biosynthetic genes, including those that encode YUCCA family flavin monooxygenases. These enzymes are essential for auxin production [168]. Conversely, auxin affects ABA levels by altering the expression of genes related to ABA production, such as NCED (9-cis-epoxycarotenoid dioxygenase) [169]. ABA can influence the transport of auxin by affecting the expression and location of auxin efflux carriers, particularly from the PIN-FORMED (PIN) family. This influences the distribution of auxin in tissues, impacting developmental processes such as root growth and lateral root formation.

In root development, the opposing roles of ABA and auxin are clear. Auxin encourages root growth and lateral root initiation by promoting cell division and expansion in root meristems. However, during drought or salt stress, high ABA levels slow down these processes [170]. ABA limits auxin signaling by decreasing auxin accumulation in the root tips and reducing the expression of auxin-responsive genes. This restriction helps conserve energy and water [171]. In *Arabidopsis*, ABA has been shown to inhibit the formation of lateral roots by lowering the expression of PIN1 and PIN2, which leads to reduced auxin movement to lateral root primordia [171]. Seed germination is another process closely controlled by the interaction between ABA and auxin. ABA promotes seed dormancy by activating transcriptional repressors including ABI3, ABI4, and ABI5 [172]. In contrast, auxin typically encourages germination by counteracting ABA signals [173]. This interaction also affects stomatal development and function. Auxin and ABA work together to regulate gas exchange and water loss. ABA triggers stomatal closure during drought, while auxin helps promote stomatal opening and guard cell expansion when conditions are favorable. ABA can inhibit auxin signaling in guard cells by suppressing the expression of auxin-responsive genes or disrupting auxin transport, leading to stomatal closure [105].

### 5.4. ABA–Cytokinin Crosstalk

ABA and cytokinins are two important phytohormones that often have opposing effects on plant growth and stress responses. At the biosynthetic level, ABA and cytokinin can influence each other. During abiotic stress conditions such as drought, ABA production increases through the NCED pathway [105]. In contrast, cytokinin production usually declines, particularly due to the repression of isopentenyl transferase (*IPT*) genes, which are crucial for cytokinin biosynthesis [174]. On the other hand, under optimal growing conditions, cytokinins boost their production and can reduce ABA levels by lowering NCED expression or increasing ABA breakdown [175]. This back-and-forth regulation helps fine-tune the balance between ABA and cytokinin based on environmental factors. When ABA signaling is dominant, it helps the plant survive stress; when cytokinins are dominant, they encourage growth in good environments.

Root and shoot development are also very sensitive to the interaction between ABA and cytokinin. ABA slows down primary root growth and encourages lateral root formation when stress occurs [176]. In contrast, cytokinins usually inhibit lateral root initiation and support the maintenance and growth of the shoot meristem [177].

ABA speeds up senescence and promotes the breakdown of chlorophyll during drought or aging, while cytokinins help delay senescence and maintain chloroplasts. Transcriptomic studies show that ABA activates genes associated with senescence (SAGs), such as *SAG12* and *ORE1*, while cytokinins inhibit these genes’ expression [178]. New evidence also highlights interactions between ABA and cytokinin signaling at the epigenetic level. ABA often encourages repressive chromatin marks (e.g., H3K27me3) at growth-related genes [179], while cytokinin promotes active chromatin marks (e.g., H3K4me3), especially in genes associated with meristem activity [180]. This kind of epigenetic regulation plays a key role in shaping long-term developmental paths in response to environmental signals. Additionally, protein stability is vital to the dynamics of ABA–cytokinin interactions. In stressful conditions, ABA signaling components such as ABI5 are stabilized, whereas cytokinin promotes the degradation of ABI5 through specific E3 ubiquitin ligases [181].

### 5.5. ABA–BR and BR–GA Interactions

**Figure 7:**
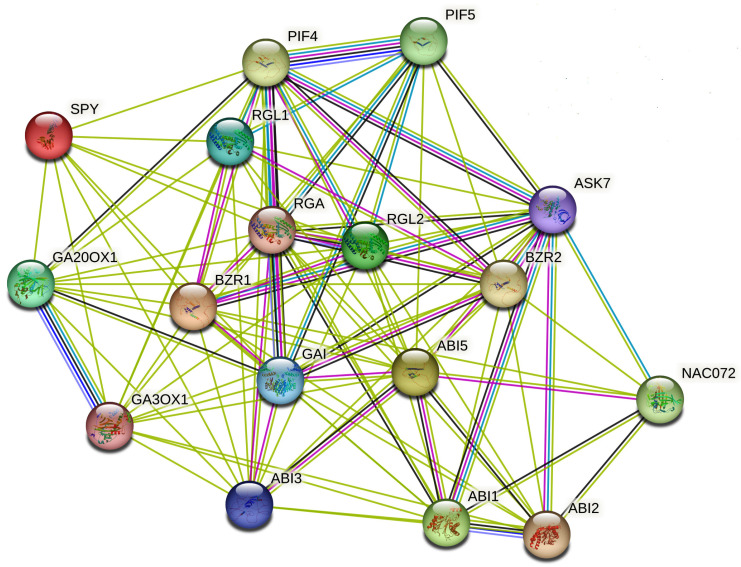
STRING analysis detecting protein–protein interaction dynamics of ABA, BRs, and GAs mapped with respect to the *Arabidopsis* database containing 17 nodes connected by 103 edges, with a PPI enrichment of *p* < 1.0 × 10^−16^ and an average node degree of 12.1. Network nodes represent proteins, colored nodes indicate query proteins and the first shell of interactors, white nodes indicate the second shell of interactors, empty nodes indicate proteins of unknown 3D structure, and filled nodes indicate the 3D structure protein of known or predicted structures. Colored lines represent various types of evidence supporting interactions. Green shows gene neighborhood, red indicates gene fusion, and blue reflects gene co-occurrence across genomes. Pink lines represent experimental evidence, light blue denotes curated database information, black shows co-expression, yellow indicates text mining, and lilac suggests protein homology. The thickness of the lines corresponds to confidence levels, with thicker lines indicating stronger evidence.

ABA, BRs, and GAs are key hormones in plants. They help manage various developmental processes and responses to environmental changes. These hormones interact through complex signaling networks, where shared components and feedback loops shape the physiological outcomes. The relationship between ABA, BRs, and GAs involves intricate interactions among multiple regulatory genes that control plant growth and stress responses (Figure 7). In the ABA–BR pathway, ABA usually inhibits growth, while BR encourages it. This opposing effect is mediated by components such as ABI1, ABI2 (PP2C phosphatases), and SnRK2 kinases, which inhibit BR signaling by phosphorylating transcription factors BES1 and BZR1 [182]. BIN2, a significant GSK3-like kinase, plays a crucial role by phosphorylating both BR regulators (BES1/BZR1) and ABA-responsive factors including ABI5, thus integrating signals from stress and growth [183]. RD26, which ABA induces, interacts with BES1 to reduce BR-driven growth, while ABA effectors ABI3 and ABI5 strengthen stress responses [184].

In interactions between BR and GA, both hormones typically promote growth. Their cooperation involves the degradation of DELLA proteins such as GAI, RGA, RGL1, and RGL2, which negatively regulate GA signaling [185]. GA biosynthetic enzymes GA20ox and GA3ox raise GA levels, enabling DELLA degradation via the GA receptor GID1 [186]. This degradation allows transcription factors such as BZR1, BES1, and PIFs (PIF4 and PIF5) to encourage the expression of growth-related genes [187]. BIN2 also indirectly aids DELLA buildup when BR signaling is absent, thus inhibiting GA responses [188]. Consequently, the flexible regulation of these signaling networks enables plants to balance growth with stress adaptation through coordinated hormonal signaling.

## 6. Applying Hormonal Crosstalk Research, Specifically in Relation to SDGs

Two important SDGs of the UN, SDG 2 (zero hunger) and SDG 13 (climate action), may be achieved by comprehending and managing hormonal crosstalk in plants (Table S2). Plant responses to a range of abiotic and biotic stressors are coordinated by phytohormones, which include GAs, ethylene, auxins, cytokinins, SA, JA, and ABA. These hormones interact, or “crosstalk,” to enable plants to adjust their defensive and developmental mechanisms in response to changing environmental circumstances [189]. Hormonal crosstalk research is essential to enhancing agricultural output stability and stress resilience in line with SDG 2, which aims to end hunger and achieve food security through sustainable agriculture. For instance, under drought stress, the antagonistic interaction between GAs and ABA regulates seed germination and dormancy, enabling crops to modify their developmental schedules in response to water availability. It is evident that several ABA and GA regulators, both positive and negative, affect germination and abiotic stress directly or indirectly. The delicate balance of bioactive ABA and GA levels is influenced by numerous transcription factors and signaling elements of these two phytohormones [118]. Likewise, SA and JA work in concert to enhance induced systemic resistance (ISR) and systemic acquired resistance (SAR), enabling plants to fend off a range of diseases while continuing to grow [190]. By identifying the essential genes and transcription factors involved in these hormone networks, advances in genomic and transcriptomic technologies have made it possible to create stress-tolerant cultivars through transgenic techniques, gene editing (such as CRISPR-Cas), and marker-assisted selection [191]. Hormonal crosstalk research encourages the creation of climate-resilient agricultural systems in line with SDG 13, which demands swift action to address climate change and its effects [192]. Rising global temperatures, unpredictable rainfall patterns, saline intrusion, and increased pest pressures all require robust plant responses. Signaling in response to salt stress and drought is significantly influenced by ABA, which also regulates stomatal closure, osmoprotectant synthesis, and root architecture. Its interaction with ethylene and JA can alter tolerance levels by altering antioxidant defense mechanisms and cellular metabolism. Hormone control also supports phenotypic plasticity, which allows a plant to adapt to a variety of agroecological zones. Crops with exact hormonal balances can now be designed thanks to modern biotechnology techniques. For instance, altering the expression of important regulators such as DREB, NAC, and WRKY transcription factors might enhance resistance to pathogens and drought while also controlling hormone-responsive pathways [193]. Additionally, systems biology techniques that integrate modeling, machine learning, and omics data are shedding light on how hormone crosstalk reacts in challenging field conditions.

Understanding hormonal crosstalk is also crucial for promoting sustainable farming practices, such as the use of biostimulants that change plant hormone levels and plant growth-promoting rhizobacteria (PGPR), which reduce dependency on artificial agrochemicals [194]. Goals for food security and reducing agricultural emissions, which lessen the effects of climate change, are both in line with this. Publications state that research on hormonal crosstalk offers a convincing framework for developing resilient and productive crops that align with the dual objectives of SDG 13 (improving climate resilience) and SDG 2 (ensuring food and nutrition security). Converting scientific discoveries into scalable agricultural technologies will require sustained investments in multidisciplinary research, policy integration, and farmer education.

## 7. Conclusions

In conclusion, this review emphasizes the critical role of phytohormones and their interactions in regulating plant responses to a wide range of abiotic and biotic stresses, with direct implications for achieving the Sustainable Development Goals, particularly SDG 2 (Zero Hunger), SDG 12 (Responsible Consumption and Production), and SDG 13 (Climate Action). Plants can sense, respond, and adapt to a variety of environmental stressors via complex hormonal signaling networks that include ABA, SA, JA, ethylene, auxins, cytokinins, and GAs. Exogenous delivery of phytohormones has emerged as a feasible and scalable technique for mitigating stress effects in crops, improving growth, antioxidant defense, and total yield performance under adverse conditions. Simultaneously, advancements in genomics, transcriptomics, and proteomics are revealing the intricate linkages and regulatory nodes of hormone pathways, providing excellent targets for biotechnological interventions. Modulating important transcription factors (e.g., MYC2, DREB, WRKY) and signaling proteins with CRISPR/Cas or other gene-editing technologies holds significant potential for developing climate-resilient crops.

## 8. Future Prospects

To effectively utilize plant hormones in achieving the Sustainable Development Goals (SDGs), future research must address critical knowledge gaps in hormonal interaction. The fine-scale spatial and temporal dynamics of hormone activity under concurrent biotic and abiotic stresses, downstream signalling, and receptor sensitivity are all crucial subjects to comprehend. Accurate and consistent crop resilience strategies will be developed with a greater understanding of these interactions. Technological advancements including systems biology, integrated omics, and AI-based modeling make it possible to map extensive stress-response networks. These methods can help with stress predictions and precision breeding by identifying key regulatory hubs within hormone pathways. Rapid field decisions and dynamic crop management are made possible by artificial intelligence and machine learning, which also aid in real-time phenotyping and predictive analytics. One of the most innovative avenues for the future is the development of precision hormone delivery devices. Traditional hormone application techniques, such as foliar sprays or soil additions, usually have drawbacks, including environmental losses, deterioration, and off-target impacts. New nanocarriers and biopolymer-based encapsulation methods are being developed to enable the regulated, environment-responsive release of phytohormones. These intelligent delivery platforms directly assist SDGs 12 (Responsible Consumption and Production) and 13 (Climate Action) by lowering resource input and environmental impact. Bringing hormonal knowledge to the field is essential to bridging the lab-to-land gap. Many promising hormone-based treatments have yet to demonstrate efficacy across a range of agroclimatic zones. An urgent need exists for large-scale, multi-location field trials that consider crop variety, soil type, and local climate variability. Collaborations between academics, farmers, and policymakers can ensure successful scaling in accordance with SDG 2 (Zero Hunger) and SDG 17 (Partnerships for the Goals). Another fascinating field is the gene editing of hormone pathways using CRISPR/Cas. By changing genes related to hormone biosynthesis, perception, or signalling, scientists can create crops that are more resilient to heat, diseases, salinity, and drought. For example, rice lines with stronger ABA signalling pathways and CRISPR editing have shown improved drought resistance and yield retention. Because gene editing offers precision and is more generally tolerated under evolving regulatory frameworks than conventional transgenic techniques, it has the potential to be utilized in agriculture in a sustainable manner. Artificial intelligence is transforming how we think about and approach hormonal therapies. Real-time crop responses to hormone treatments can be monitored using AI-driven platforms that make use of drones, sensors, and imaging technologies. Additionally, gene targets for stress tolerance can be predicted using AI-integrated multi-omics data analysis. These discoveries are critical to the creation of adaptable, site-specific hormone control strategies. It will be necessary to integrate phytohormonal insights with smart technologies and sustainable practices in order to build climate-resilient agriculture in the future. Hormone-based innovations backed by translational research and governmental assistance must be deliberately implemented to achieve food security and environmental sustainability. In a world where food instability and climate change are becoming more common, plant hormone networks provide a revolutionary tool. The transition from hormones to harvests is therefore an essential step in accomplishing the Sustainable Development Goals.

## Figures and Tables

**Table 1:** KEGG pathway enrichment of major hormonal cross-talk.

Term ID	Term Description	Observed Gene Count	Background Gene Count	Strength	Signal	False Discovery Rate	Matching Proteins
ath04075	Plant hormone signal transduction	32	289	1.75	8.09	2.40 × 10^−16^	COI1, ABI2, BRI1, EIN3, ARR10, TGA2, ETR1, ABI1, NPR1, ARF7, CTR1, PR1, SRK2D, SRK2I, MYC2, TGA1, RGL2, ERF1B, BZR1, TIFY9, BAK1, AUX1, AHK3, AHK2, TIFY10A, BZR2, GAI, ABF2, GID1A, EIN2, ARR5, RGA
ath04016	MAPK signaling pathway-plant	13	136	1.69	4	1.25 × 10^−16^	ABI2, EIN3, ETR1, ABI1, CTR1, PR1, SRK2D, SRK2I, MYC2, ERF1B, BAK1, EIN2, ACS6
ath00904	Diterpenoid biosynthesis	3	22	1.84	1.05	0.00072	GA3OX1, GA20OX1, GA2OX2
ath01110	Biosynthesis of secondary metabolites	11	1219	0.66	0.6	0.00075	CYP90B1, GA3OX1, GA20OX1, ACO1, CYP90A1, IPT3, CYP74A, OPR3, NCED3, ACS6, GA2OX2
ath00905	Brassinosteroid biosynthesis	2	8	2.1	0.81	0.0045	CYP90B1, CYP90A1
ath00380	Tryptophan metabolism	3	62	1.39	0.7	0.0064	YUC6, TAA1, YUC2
ath01100	Metabolic pathways	13	2285	0.46	0.39	0.0076	CYP90B1, GA3OX1, GA20OX1, ACO1, CYP90A1, YUC6, IPT3, CYP74A, OPR3, NCED3, TAA1, ACS6, YUC2

## Supplementary Materials

The following supporting information can be downloaded at: https://www.mdpi.com/article/10.3390/plants14152322/s1, Figure S1: k-means functional clustering of proteins involved in hormonal cross talk mechanism [K-means clustering (k = 6) was applied to group functionally related proteins, with each cluster visualized in a distinct color]; Table S1: List of genes, their abbreviations, and functional descriptions; Table S2: Plant hormones and Sustainable Development Goal Alignment.
