# Supplementary material for: From Hormones to Harvests: A Pathway to Strengthening Plant Resilience for Achieving Sustainable Development Goals
Source: Plants (Basel). 2025 Jul 27;14(15):2322. doi: 10.3390/plants14152322 (PMC12348992; doi:10.3390/plants14152322)
Supplement: Supplementary file 1 [file plants-14-02322-s001.zip › plants-3751658 FigureS1.pdf]

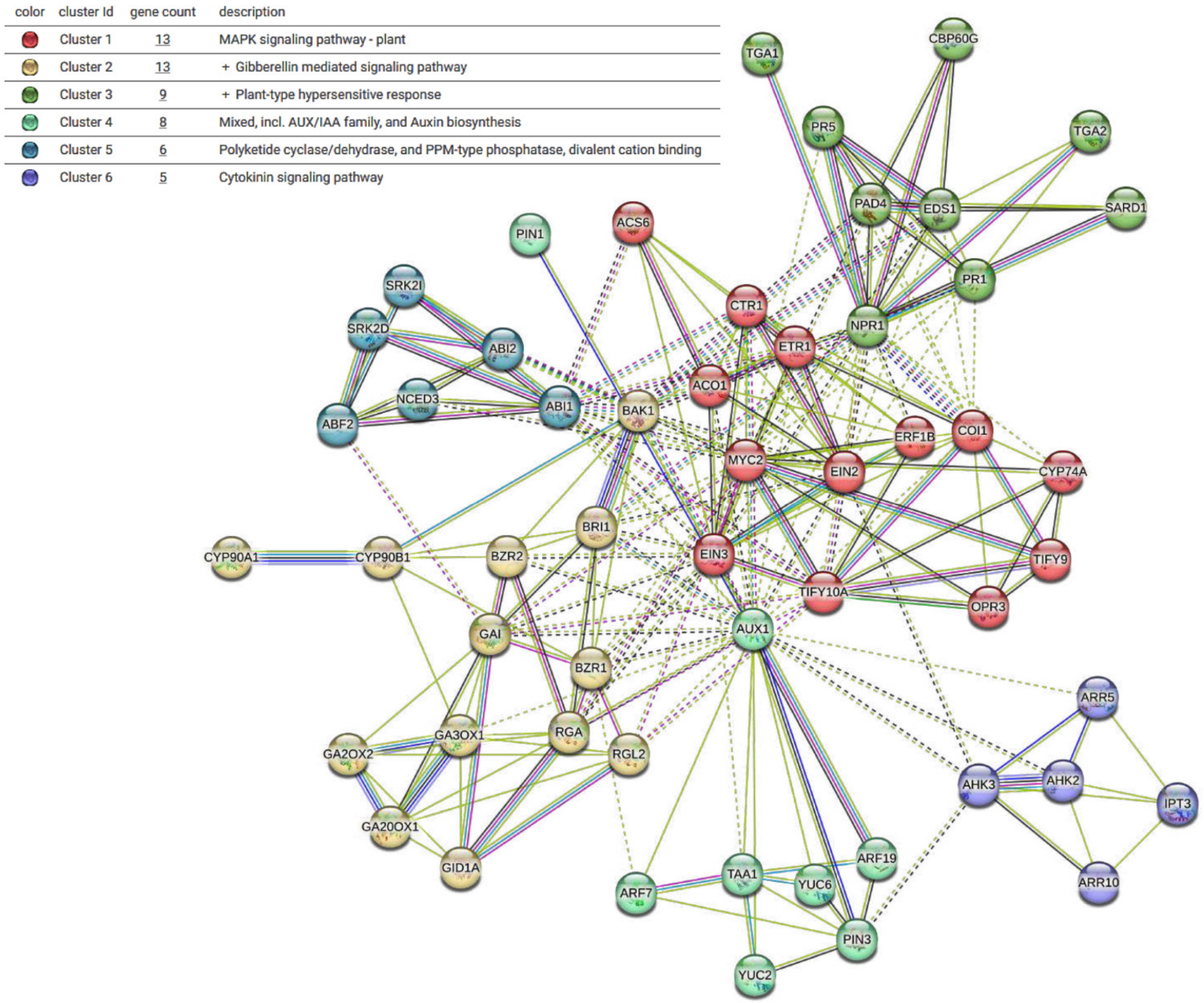

Figure S1. k-means functional clustering of proteins involved in hormonal cross talk mechanism [K-means clustering (k = 6) was applied to group functionally related proteins, with each cluster visualized in a distinct color]
