# Supplementary material for: From Hormones to Harvests: A Pathway to Strengthening Plant Resilience for Achieving Sustainable Development Goals
Source: Plants (Basel). 2025 Jul 27;14(15):2322. doi: 10.3390/plants14152322 (PMC12348992; doi:10.3390/plants14152322)
Supplement: Supplementary file 1 [file plants-14-02322-s001.zip › plants-3751658 TableS1.pdf]

Table S1. List of genes, their abbreviations, and functional descriptions

Gene	Full Name / Abbreviation	Function
**Abscisic Acid Signaling**		
NCED3	9-cis-Epoxycarotenoid Dioxygenase 3	Key enzyme in ABA biosynthesis
PYL4, PYL5, PYL8	Pyrabactin Resistance-Like Proteins	ABA receptors, initiate ABA signal transduction
SnRK2.2, SnRK2.3, SnRK2.6 (OST1)	SNF1-Related Protein Kinases	Activate ABA responses like stomatal closure
ABI1, ABI2	ABA-Insensitive 1/2 (PP2C phosphatases)	Negative regulators of ABA signaling
AREB1, ABF2	ABA-Responsive Element Binding Protein 1 / ABRE-Binding Factor 2	ABA-responsive transcription factors
**Salicylic Acid Signaling**		
ICS1 (SID2)	Isochorismate Synthase 1	Catalyzes SA biosynthesis
EDS1	Enhanced Disease Susceptibility 1	Regulates SA accumulation and signaling
PAD4	Phytoalexin Deficient 4	Works with EDS1 to amplify SA responses
NPR1	Nonexpresser of PR Genes 1	Master regulator of SA-mediated defense genes
TGA1, TGA2	TGA Transcription Factors	Interact with NPR1 to activate PR genes
PR1, PR2, PR5	Pathogenesis-Related Genes	Marker genes for systemic acquired resistance (SAR)
**Jasmonic Acid Signaling**		
AOS	Allene Oxide Synthase	Enzyme in JA biosynthesis pathway
OPR3	12-Oxophytodienoate Reductase 3	JA biosynthesis enzyme
COI1	Coronatine-Insensitive 1	JA receptor component
JAZ1, JAZ10	Jasmonate ZIM-Domain Proteins	JA signaling repressors
MYC2	MYC Transcription Factor 2	Activator of JA-responsive genes
**Ethylene Signaling**		
ACS6	1-Aminocyclopropane-1-Carboxylic Acid Synthase 6	Enzyme in ethylene biosynthesis
ACO1	ACC Oxidase 1	Converts ACC to ethylene
ETR1	Ethylene Response 1	Ethylene receptor
CTR1	Constitutive Triple Response 1	Negative regulator of ethylene signaling
EIN2, EIN3	Ethylene Insensitive 2/3	Positive regulators of ethylene responses
**ERF1**	Ethylene Response Factor 1	Activates ethylene-responsive genes

**Cytokinin Signaling**		
IPT3	Isopentenyl Transferase 3	Involved in cytokinin biosynthesis
AHK2, AHK3	Arabidopsis Histidine Kinases	Cytokinin receptors
ARR5 (Type-A)	Arabidopsis Response Regulator 5	Negative feedback regulator of CK signaling
ARR1, ARR10 (Type-B)	Arabidopsis Response Regulators	Transcription factors in cytokinin signaling
**Brassinosteroid Signaling**		
DWF4, CPD	DWARF4, Constitutive Photomorphogenesis and Dwarfism	BR biosynthesis enzymes
BRI1	Brassinosteroid Insensitive 1	BR receptor
BAK1	BRI1-Associated Kinase 1	Co-receptor with BRI1
BZR1, BES1	BR-Insensitive 1-EMS Suppressor 1	Transcription factors in BR signaling
CYP90A1, CYP90B1	Cytochrome P450 Monooxygenases	Catalyze steps in BR biosynthesis
**Auxin Signaling**		
TAA1	Tryptophan Aminotransferase of Arabidopsis 1	Converts Trp to IPA, a step in auxin biosynthesis
YUC2, YUC6	YUCCA Flavin Monooxygenases	Catalyze auxin biosynthesis
AUX1	Auxin Resistant 1	Auxin influx carrier
PIN1, PIN3	PIN-Formed Proteins	Auxin efflux transporters
ARF7, ARF19	Auxin Response Factors	Transcription factors in auxin signaling
**Gibberellin Signaling**		
GA20ox1, GA3ox1	GA 20-/3-Oxidases	Enzymes in active GA biosynthesis
GA2ox2	GA 2-Oxidase 2	GA deactivating enzyme
GID1A	GA Insensitive Dwarf 1A	GA receptor
GAI, RGA, RGL2	DELLA Proteins	Negative regulators of GA signaling
