# Supplementary material for: From Hormones to Harvests: A Pathway to Strengthening Plant Resilience for Achieving Sustainable Development Goals
Source: Plants (Basel). 2025 Jul 27;14(15):2322. doi: 10.3390/plants14152322 (PMC12348992; doi:10.3390/plants14152322)
Supplement: Supplementary file 1 [file plants-14-02322-s001.zip › plants-3751658 TableS2.pdf]

Table S2. Plant hormones and Sustainable Development Goal Alignment

Plant Hormone(s)	Key Functions	Relevant SDG(s)
Auxins & Cytokinins	Control nutrient intake, organogenesis, and root/shoot development	SDG 2 (Zero Hunger), SDG 15 (Life on Land)
Abscisic Acid (ABA)	Enhances resistance to salinity and drought stress by regulating genes and stomatal closure.	SDG 13 (Climate Action), SDG 6 (Clean Water & Sanitation)
Gibberellins (GA)	Encourage flowering, stem elongation, and seed germination	SDG 2 (Zero Hunger)
Ethylene	Mediates responses to biotic and abiotic stress, regulates fruit ripening and senescence	SDG 12 (Responsible Consumption), SDG 9 (Industry Innovation)
Jasmonic Acid (JA)	Defense against herbivores/pathogens; crosstalk with salicylic acid	SDG 15 (Life on Land), SDG 3 (Good Health and Well-being)
Salicylic Acid (SA)	Induces systemic acquired resistance (SAR) against pathogens Infection	SDG 2 (Zero Hunger), SDG 15 (Life on Land)
Brassinosteroids (BR)	Enhance plant growth, stress resistance, and development	SDG 2(Zero Hunger), SDG 13(Good Health and Well-being)
Strigolactones	Regulate shoot branching and root architecture; mediate symbiosis with mycorrhiza	SDG 2 (Zero Hunger), SDG 15 (Life on Land)
